# Causal Association Between Antidepressant Use and Thyroid Disease Risk: A Two-Sample Mendelian Randomization Study

**DOI:** 10.62641/aep.v54i4.2313

**Published:** 2026-08-15

**Authors:** Yingxian Ling, Qisong Chen

**Affiliations:** ^1^Department of Pharmacy, Ningbo Rehabilitation Hospital, 315040 Ningbo, Zhejiang, China; ^2^Department of Pharmacy, Ningbo Hospital of Integrated Traditional Chinese and Western Medicine, 315300 Ningbo, Zhejiang, China

**Keywords:** antidepressants, Hashimoto’s thyroiditis, hypothyroidism, Mendelian randomisation, GWAS

## Abstract

**Background:**

Observational studies have reported associations between antidepressant use and thyroid dysfunction; however, confounding precludes firm causal inference. This study aimed to evaluate the potential causal association between genetically predicted antidepressant use and the risks of Hashimoto’s thyroiditis, hypothyroidism and thyroid cancer using Mendelian randomisation (MR).

**Methods:**

Publicly available genome-wide association study (GWAS) summary statistics were used, with genetically predicted antidepressant use as the exposure and Hashimoto’s thyroiditis, hypothyroidism and thyroid cancer as the outcomes. Following data harmonisation, 13 single nucleotide polymorphisms (SNPs) meeting the prespecified significance threshold were selected as instrumental variables. The mean F statistic was 24.12 (range, 20.96–29.98), indicating that weak-instrument bias was unlikely. Inverse variance weighted (IVW) was used for the primary analysis, with MR-Egger regression, weighted-median and mode-based methods used in sensitivity analyses. Cochran ’s Q test was used to assess heterogeneity, and the MR-Egger intercept was used to assess directional horizontal pleiotropy.

**Results:**

Genetically predicted antidepressant use was positively associated with the risk of Hashimoto’s thyroiditis (IVW: odds ratio [OR] = 1.483, 95% confidence interval [CI]: 1.176–1.871; *p* = 0.001). A weak but statistically significant positive association was also observed for hypothyroidism (IVW: OR = 1.012, 95% CI: 1.002–1.023; *p* = 0.025). The directions of effect were generally consistent across sensitivity analyses. No evidence of an association with thyroid cancer was identified (IVW: *p* = 0.407). No evidence of bias was found in the heterogeneity test and the horizontal pleiotropy (*p* > 0.05), and the leave-one-out analysis confirmed the robustness of the results.

**Conclusions:**

This study provides genetic evidence of potential associations between antidepressant use and increased risks of Hashimoto’s thyroiditis and hypothyroidism. These findings support consideration of thyroid-function monitoring in patients receiving antidepressant treatment, particularly those with pre-existing thyroid risk factors.

## Introduction

Depression is a leading contributor to the global burden of mental disorders, with an estimated lifetime prevalence of approximately 15–18% [1] and an increasing prevalence among younger people. Over the past two decades, antidepressant prescribing has continued to increase worldwide, and more than 13% of adults in the United States use antidepressants [2]. Beyond their well-recognised adverse effects, including gastrointestinal symptoms, hepatotoxicity, cardiovascular disturbances, sexual dysfunction, hyponatraemia and reduced bone mineral density [3, 4], their potential effects on the endocrine system remain poorly characterised. In particular, causal evidence regarding their effects on thyroid function is limited.

The clinical spectrum of thyroid disease encompasses autoimmune inflammation, functional disorders and neoplasia. Hashimoto’s thyroiditis is the most common organ-specific autoimmune disease and the principal cause of hypothyroidism [5], whereas thyroid cancer, the most common endocrine malignancy, arises through molecular drivers distinct from autoimmune mechanisms [6]. These three conditions therefore represent biologically distinct outcomes for evaluating the potential effects of antidepressant use.

The clinical association between depression and thyroid dysfunction is well documented. Patients with major depression often exhibit abnormalities of the hypothalamic–pituitary–thyroid (HPT) axis and elevated thyroid autoantibody levels [7, 8]. Observational studies have also reported a high prevalence of thyroid dysfunction among patients with depression, including those who have not received antidepressant treatment [9, 10]. A direct pharmacological effect is also biologically plausible. Selective serotonin reuptake inhibitors (SSRIs) act through the serotonergic system, which contributes to regulation of the HPT axis [11]; serotonin receptors and transporters are expressed in both thyroid tissue [12] and immune cells [13, 14]. Furthermore, fluctuations in thyroid-stimulating hormone (TSH) levels and thyroid autoantibody titres have been observed during treatment [15]. However, because depression itself disrupts the HPT axis and is associated with chronic inflammation, metabolic disturbances and lifestyle changes [9], conventional observational designs cannot reliably distinguish drug-related effects from confounding by indication.

Mendelian randomisation (MR) addresses some of these limitations by using genetic variants that are randomly allocated at meiosis and are generally less susceptible to postnatal confounding and reverse causation as instrumental variables [16]. MR has increasingly been used to investigate potential causal relationships between exposures and disease outcomes. Previous MR studies have examined associations between psychiatric exposures, including depression, and outcomes such as osteoporosis and cardiovascular disease, thereby providing an indirect basis for evaluating antidepressant-related adverse outcomes [17, 18]. However, MR evidence concerning antidepressant use and thyroid disease remains scarce. Given the increasing use of antidepressants, including among middle-aged and older women who are at increased risk of thyroid disease, clarification of this relationship has clinical and public-health relevance.

We therefore conducted a two-sample MR study using publicly available, large-scale genome-wide association study (GWAS) summary data. Genetically predicted antidepressant use was evaluated as the exposure, and Hashimoto’s thyroiditis, hypothyroidism and thyroid cancer were evaluated as outcomes, with the aim of providing genetic evidence to inform thyroid-function monitoring during antidepressant treatment.

## Materials and Methods

### Study Design

This two-sample MR study used published GWAS summary statistics to evaluate potential causal associations between genetically predicted antidepressant use and the risks of Hashimoto’s thyroiditis, hypothyroidism and thyroid cancer. MR analysis relies on three core assumptions: (1) the instrumental variables are strongly associated with the exposure; (2) the instrumental variables are independent of confounders of the exposure–outcome association; and (3) the instrumental variables affect the outcome only through the exposure, with no alternative direct pathway.

### Exposure Data

The exposure was genetically predicted antidepressant use (OpenGWAS ID: ebi-a-GCST90018998), derived from the European-ancestry GWAS reported by Sakaue *et al*. [19]. In Supplementary Materials provided of that study, medication-use phenotypes are listed only for the UK Biobank and not for FinnGen; this dataset therefore represents a single-cohort UK Biobank GWAS and does not include FinnGen participants. The UK Biobank GWAS of antidepressant use (Anatomical Therapeutic Chemical code N06A) comprised 33,757 cases and 270,405 controls (total N = 304,162).

### Outcome Data

The outcomes comprised three thyroid disorders. Summary statistics for Hashimoto’s thyroiditis (OpenGWAS ID: ebi-a-GCST90018855) and thyroid cancer (OpenGWAS ID: ebi-a-GCST90018929) were also obtained from Sakaue *et al*. [19]. Unlike the antidepressant-use exposure, these disease endpoints were derived from European trans-biobank meta-analyses combining the UK Biobank and FinnGen cohorts (Supplementary Materials provided of Sakaue *et al*. [19]). For Hashimoto’s thyroiditis, the meta-analysis included 220 cases and 339,611 controls from the UK Biobank and 15,434 cases and 40,375 controls from FinnGen (total N = 395,640). As documented in Supplementary Materials provided of Sakaue *et al*. [19], Hashimoto’s disease (International Classification of Diseases, 10th Revision [ICD-10] E06.3; phecode 245.21) and hypothyroidism (ICD-10 E03; phecode 244.4) map to the same FinnGen endpoint (HYPOTHYROIDISM). Consequently, the FinnGen component of this GWAS (15,434 cases and 40,375 controls) comprises general hypothyroidism cases rather than diagnoses specific to Hashimoto’s disease, whereas the UK Biobank component (220 cases; phecode 245.21) is specific to Hashimoto’s disease. For thyroid cancer, the meta-analysis included 553 cases and 355,783 controls from the UK Biobank and 501 cases and 135,137 controls from FinnGen (total N = 491,974).

Summary statistics for hypothyroidism were obtained from the dataset generated by Loh *et al*. (2018) [20] using Bayesian-Optimized Linear mixed Model (BOLT-LMM) in the UK Biobank cohort (OpenGWAS ID: ebi-a-GCST90029022). Although OpenGWAS records a sample size of 473,703 for this dataset, the effective analytical sample documented in Supplementary Materials provided of Loh *et al*. [20] comprised 459,324 individuals of European ancestry, with a case fraction of 0.049, corresponding to approximately 22,507 cases and 436,817 controls. Unlike conventional GWAS pipelines that restrict analyses to unrelated individuals, BOLT-LMM retains all genotyped participants, including close relatives, to maximise statistical power. The validity of BOLT-LMM *p* values for binary traits at this case fraction (4.9%) was confirmed by simulations in the original study, and each of the 111 genome-wide significant loci identified for hypothyroidism contained a lead single nucleotide polymorphism (SNP) with a minor allele frequency >1% (Supplementary Note, Section 6, and Supplementary Materials provided of Loh *et al*. [20]).

All exposure and outcome datasets comprised populations of European ancestry. Detailed definitions, OpenGWAS IDs and sample sizes are summarised in Table 1 (Ref. [19, 20]).

**Table 1.** **Exposure and outcome variable data source information**.

Expose/Outcome	OpenGWAS ID	Year	Population	Sample size	Case	Control
Medication use (antidepressants)	ebi-a-GCST90018998 [19]	2021	European	304,162	33,757	270,405
Hashimoto’s thyroiditis	ebi-a-GCST90018855 [19]	2021	European	395,640	15,654	379,986
Thyroid cancer	ebi-a-GCST90018929 [19]	2021	European	491,974	1054	490,920
Hypothyroidism	ebi-a-GCST90029022 [20]	2018	European	459,324	22,507	436,817

Note: Summary statistics for Hashimoto’s thyroiditis, thyroid cancer and antidepressant use were derived from Sakaue *et al*. [19]. As documented in Supplementary Materials provided of Sakaue *et al*. (2021) [19], antidepressant use (ATC code N06A; OpenGWAS ID: ebi-a-GCST90018998) was analysed in the UK Biobank cohort only (33,757 cases and 270,405 controls), because medication-use phenotypes were unavailable in FinnGen. Hashimoto’s thyroiditis (OpenGWAS ID: ebi-a-GCST90018855) and thyroid cancer (OpenGWAS ID: ebi-a-GCST90018929) represent European meta-analyses combining the UK Biobank and FinnGen cohorts (Hashimoto’s thyroiditis: 15,654 cases and 379,986 controls; thyroid cancer: 1054 cases and 490,920 controls). Summary statistics for hypothyroidism (OpenGWAS ID: ebi-a-GCST90029022) were derived from Loh *et al*. (2018) [20]. As reported in Supplementary Materials provided of that study, the effective analytical sample comprised 459,324 individuals of European ancestry (case fraction = 0.049; approximately 22,507 cases and 436,817 controls). The number of hypothyroidism cases was estimated as N × the case fraction because stratified case and control numbers were not reported directly. In FinnGen, Hashimoto’s disease and hypothyroidism share the same endpoint (HYPOTHYROIDISM; Supplementary Materials provided of Sakaue *et al*. [19]); therefore, the case and control counts for the FinnGen component of the Hashimoto’s thyroiditis GWAS represent general hypothyroidism diagnoses.

### Statistical Analysis

#### Selection of Instrumental Variables

SNPs loci associated with antidepressant use were extracted from the exposure GWAS. conventional genome-wide significance threshold of *p* < 5 × 10^-8^ was applied for SNPs selection; however, only 1 independent SNPs satisfied this criterion, which was insufficient to perform the primary inverse variance weighted (IVW) analysis or any sensitivity analyses, given that tests such as the MR-Egger intercept test, Cochran’s Q test, and leave-one-out analysis each require a minimum of 3 SNPs. Therefore, consistent with several previously published MR studies [21, 22, 23], the threshold was relaxed to *p* < 5 × 10^-6^ to obtain sufficient instruments for comprehensive sensitivity analyses. SNPs were clumped at a linkage-disequilibrium threshold of r^2^< 0.001 within a 10,000-kb window, using European-ancestry genotype data The strength of instrumental variables is evaluated by F statistics. F statistics greater than 10 indicates that the strength of instrumental variables is sufficient, which can effectively avoid the bias of weak instrumental variables [24]. The primary Mendelian randomization analysis was performed using the IVW method, which yields the most causal effects estimate under the assumption of no horizontal pleiotropy. By default, the TwoSampleMR package employs a multiplicative random-effects model; however, since no significant heterogeneity was found in our analysis (*p* > 0.05), a fixed-effects model was effectively employed for the IVW estimation. In order to enhance the robustness of the results, MR-Egger regression, weighted median method and weighted mode method were used for supplementary analysis. MR-Egger regression can detect and correct directional pleiotropic bias. The weighted median method can provide consistent estimates even if 50% of the instrumental variables are invalid [25]. The weighted mode method was applied to identify the most common causal effect estimates.

Sensitivity analysis was undertaken to assess the robustness of the results and test Mendelian randomization hypothesis. First, Cochran’s Q statistic was calculated to evaluate the heterogeneity between SNPs loci [26]. A Q statistic *p* value below 0.05 was considered indicative of significant heterogeneity, which may stem from horizontal pleiotropy or population stratification. Second, the directional pleiotropy was evaluated by the MR-Egger intercept test. The significant deviation of the intercept from zero indicates the existence of horizontal pleiotropy, which may violate the exclusivity hypothesis of Mendelian randomization. Third, a leave-one-out analysis was used to eliminate each SNPs site one by one, and then the Mendelian randomization analysis was performed again to evaluate the impact of a single site on the overall causal estimation and identify potential abnormal driving sites. In addition, funnel plots were constructed to visually detect small sample effects and potential publication bias.

All Mendelian randomization analysis results were reported by MR results are reported as odds ratios (ORs) with 95% confidence intervals (CIs). Interpretation focused on the IVW estimates and was supported by the complementary MR methods and sensitivity analyses. All analyses were performed using R version 4.3.3 (R Foundation for Statistical Computing, Vienna, Austria), with TwoSampleMR version 0.6.10 (MRC Integrative Epidemiology Unit, University of Bristol, Bristol, UK) and its dependencies: data.table version 1.16.2, dplyr version 1.1.4, ieugwasr version 1.0.2, VariantAnnotation version 1.52.0 and gwasglue version 0.0.0.9000. All tests were two-sided, and *p* < 0.05 was considered statistically significant. Forest, scatter, funnel and leave-one-out plots were used to visualise the MR and sensitivity-analysis results. As this was an in silico study based on publicly available summary-level data, no laboratory equipment, clinical devices or experimental drugs were used.

### Ethics Statement

All data used in this study were publicly available, de-identified GWAS summary statistics. Therefore, separate ethics committee approval was not required for the present analysis. The original studies received approval from the relevant institutional review boards or ethics committees, and all participants provided written informed consent.

## Results

### Selection and Strength Assessment of Instrumental Variables

After screening the GWAS data for antidepressant use, 13 independent SNPs meeting the prespecified significance threshold were selected as instrumental variables. Their F statistics ranged from 20.96 to 29.98 (mean, 24.12), and all exceeded 10 (**Supplementary Table 1**), indicating adequate instrument strength and a low risk of weak-instrument bias. Following harmonisation, all 13 SNPs were matched across the three outcome datasets, and none was excluded because of ambiguous palindromic sequences was observed, ensuring the consistency of the causal assessment and enabling subsequent alleles.

### Statistical Results of Mendelian Randomization Analysis

Mendelian randomization analysis assessed the causal association MR analyses evaluated the associations between genetically predicted antidepressant use and the three thyroid outcomes (Table 2). For Hashimoto’s thyroiditis, the IVW analysis showed a statistically significant positive association (OR = 1.483, 95% CI: 1.176–1.871; *p* = 0.001). The weighted-median estimate was directionally consistent but did not reach statistical significance its effect direction was fully consistent with the IVW estimates, and it demonstrated a consistent positive association trend across multiple sensitivity models, thereby enhancing the credibility of the causal inference (*p* = 0.078). No statistically significant associations were observed with MR-Egger regression (*p* = 0.151), the weighted-mode method (*p* = 0.351) or the simple-mode method (*p* = 0.371).

**Table 2.** **Mendelian randomization analysis results**.

Outcome	Method	SNPs	*p*-value	OR	95% CI
Hashimoto’s thyroiditis	MR Egger	13	0.151	2.093	0.819–5.348
	Weighted median	13	0.078	1.310	0.970–1.770
	IVW	13	0.001	1.483	1.176–1.871
	Simple mode	13	0.371	1.262	0.773–2.063
	Weighted mode	13	0.351	1.258	0.791–2.001
Hypothyroidism	MR Egger	13	0.683	1.009	0.967–1.054
	Weighted median	13	0.002	1.015	1.006–1.024
	IVW	13	0.025	1.012	1.002–1.023
	Simple mode	13	0.046	1.016	1.002–1.030
	Weighted mode	13	0.067	1.015	1.000–1.030
Thyroid cancer	MR Egger	13	0.538	2.177	0.198–23.950
	Weighted median	13	0.898	0.949	0.425–2.119
	IVW	13	0.407	1.273	0.720–2.252
	Simple mode	13	0.605	0.704	0.192–2.573
	Weighted mode	13	0.628	0.730	0.211–2.524

IVW, inverse variance weighted; OR, odds ratio; 95% CI, 95% confidence interval; SNPs, single nucleotide polymorphisms; MR, Mendelian randomisation.

For hypothyroidism, the IVW analysis indicated a small positive association (OR = 1.012, 95% CI: 1.002–1.023; *p* = 0.025). The weighted-median method supported this finding (OR = 1.015, 95% CI: 1.006–1.024; *p* = 0.002), and the simple-mode estimate was also statistically significant (*p* = 0.046). The weighted-mode (*p* = 0.067) and MR-Egger (*p* = 0.683) estimates were not statistically significant.

For thyroid cancer, neither the IVW analysis (OR = 1.273, 95% CI: 0.720–2.252; *p* = 0.407) nor any complementary analysis identified evidence of an association with genetically predicted antidepressant use (all *p* > 0.4) found a causal association between genetically predicted antidepressant use and the risk of thyroid cancer).

### Forest Plot of Mendelian Randomization Analysis Results

**Fig. 1.**
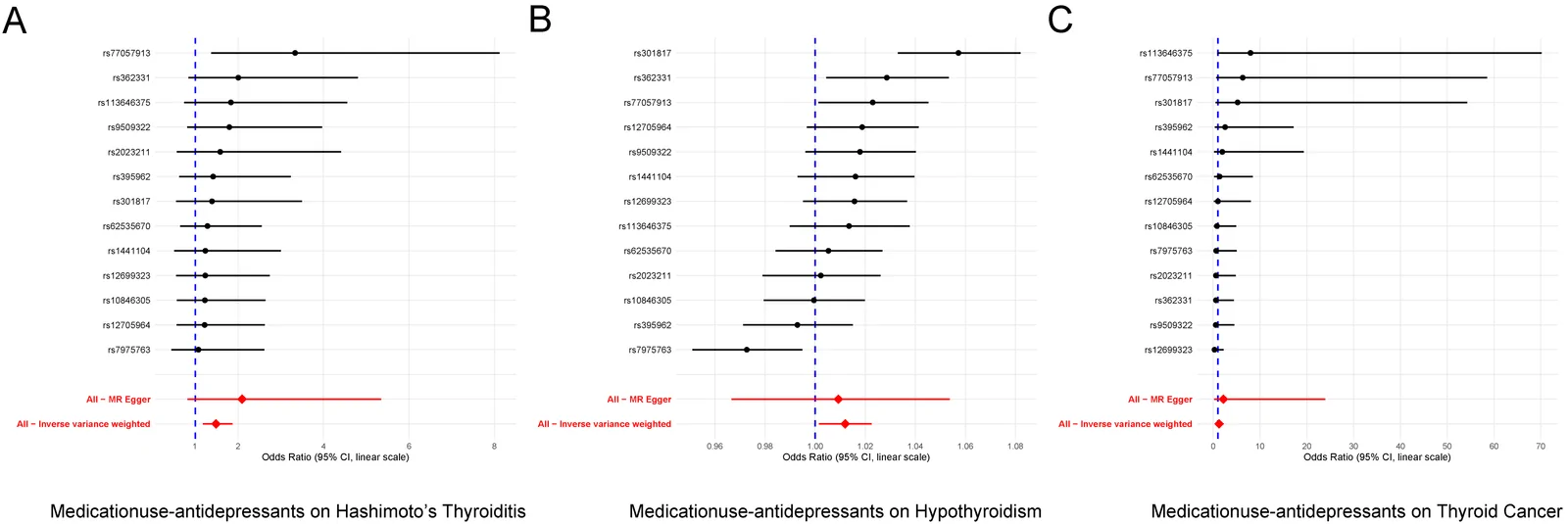
**Forest plots of MR analysis results**. (A–C) Forest plots showing the results of inverse variance weighted (IVW) and Mendelian randomisation (MR)-Egger analyses of the causal relationship between antidepressant use and Hashimoto’s thyroiditis (A), hypothyroidism (B), and thyroid cancer (C). The horizontal axis is presented on a linear scale representing the odds ratio (OR) and its 95% confidence interval (CI). Note that the 95% CIs are asymmetric on this scale, with wider intervals extending toward the upper bound, which is inherent to the exponentiation of symmetric log-scale estimates. The blue dashed line indicates the null effect (OR = 1.0), and the red diamonds represent the pooled causal estimates.

The forest plot presents the causal effect estimates and their confidence intervals derived from the various Mendelian randomization methods (Fig. 1). For Hashimoto’s thyroiditis, the forest plot demonstrated that the pooled point estimates from both the IVW method and MR-Egger regression consistently fell to the right of the null line (OR = 1.0), suggesting a potential association between antidepressant use and increased risk of Hashimoto’s thyroiditis. Notably, the confidence interval for the IVW method does not traverse the null line at all, confirming the statistical significance of this causal association based on the graphical distribution.

For hypothyroidism, the forest plot showed that the point estimates from all pooled analysis methods were positioned to the right of the null line, with comparatively narrow confidence intervals. Although the absolute magnitudes of the effect sizes were small, the confidence interval of IVW method did not cross the null line, and the graphical features supported a positive association between antidepressant use and the risk of this condition.

In contrast, the forest plot for thyroid cancer exhibits marked dispersion, with point estimates for individual SNPs widely distributed on both sides of the null line. Although the point estimates from all pooled analysis methods are slightly skewed to the right, their confidence intervals all extend significantly beyond the null line, intuitively indicating that no statistically significant causal evidence has been identified to date.

### Scatter Plots of Mendelian Randomization Analysis Results

**Fig. 2.**
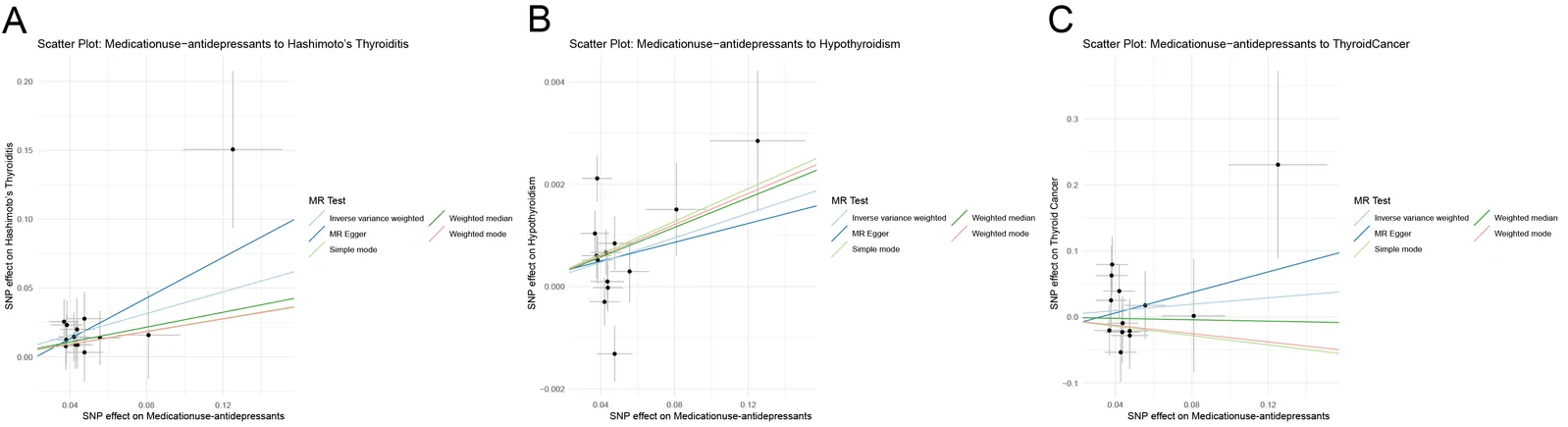
**Scatter plots of MR analysis results**. (A–C) Scatter plots showing the results of Mendelian randomisation (MR) analyses of the causal relationship between antidepressant use and Hashimoto’s thyroiditis (A), hypothyroidism (B), and thyroid cancer (C).

The scatter plots illustrate the per-SNPs effect size distributions for each SNPs locus regarding the relationship between exposure and outcome, as well as the fitted regression lines from different MR methods (Fig. 2). In the analysis of Hashimoto’s thyroiditis, the majority of instrumental variables showed a consistent positive association trend, with both the IVW method and MR-Egger regression yielding positive slopes that were consistent in direction. For hypothyroidism, the effect sizes distribution of the instrumental variables was relatively dense, and the fitted lines from all major MR methods exhibited a weak but stable positive slope. In contrast, in the analysis of thyroid cancer, the effect-size distribution across SNPs loci was markedly dispersed, and the slopes of the regression lines from different fitting methods diverged and often fell below the significance threshold, with no clear causal regression trend observed.

### Heterogeneity Assessment

Heterogeneity among SNP-specific estimates was assessed using Cochran’s Q statistic (Table 3). For Hashimoto’s thyroiditis, neither the IVW analysis (Q = 5.670, df = 12; *p* = 0.932) nor MR-Egger regression (Q = 5.118, df = 11; *p* = 0.925) indicated significant heterogeneity. Similarly, no significant heterogeneity was detected for hypothyroidism using IVW (Q = 14.481, df = 12; *p* = 0.328) or MR-Egger regression (Q = 14.431, df = 11; *p* = 0.282), or for thyroid cancer using IVW (Q = 11.206, df = 12; *p* = 0.511) or MR-Egger regression (Q = 11.002, df = 11; *p* = 0.443 further indicate consistency in the effects of the instrumental variables. Since the *p*-values for the heterogeneity tests of all outcomes were significantly greater than 0.05, this suggests that the effects of the instrumental variables on the outcomes are evenly distributed, strongly validating the robustness and reliability of the causal estimates in this study.

**Table 3.** **Mendelian randomization heterogeneity test**.

Outcome	Method	Cochran’s Q	df	*p* for heterogeneity
Hashimoto’s thyroiditis	MR Egger	5.118	11.000	0.925
	IVW	5.670	12.000	0.932
Hypothyroidism	MR Egger	14.431	11.000	0.282
	IVW	14.481	12.000	0.328
Thyroid cancer	MR Egger	11.002	11.000	0.443
	IVW	11.206	12.000	0.511

Q, Cochran ’s Q statistic; MR, Mendelian randomisation; df, degree of freedom; *p* for heterogeneity, heterogeneity test *p* value. *p* < 0.05 indicates that there is significant heterogeneity between instrumental variables. Since no significant heterogeneity was found, this study mainly used the fixed effect model for inverse variance weighted (IVW) analysis.

### Funnel Plot of Mendelian Randomization Analysis Results

**Fig. 3.**
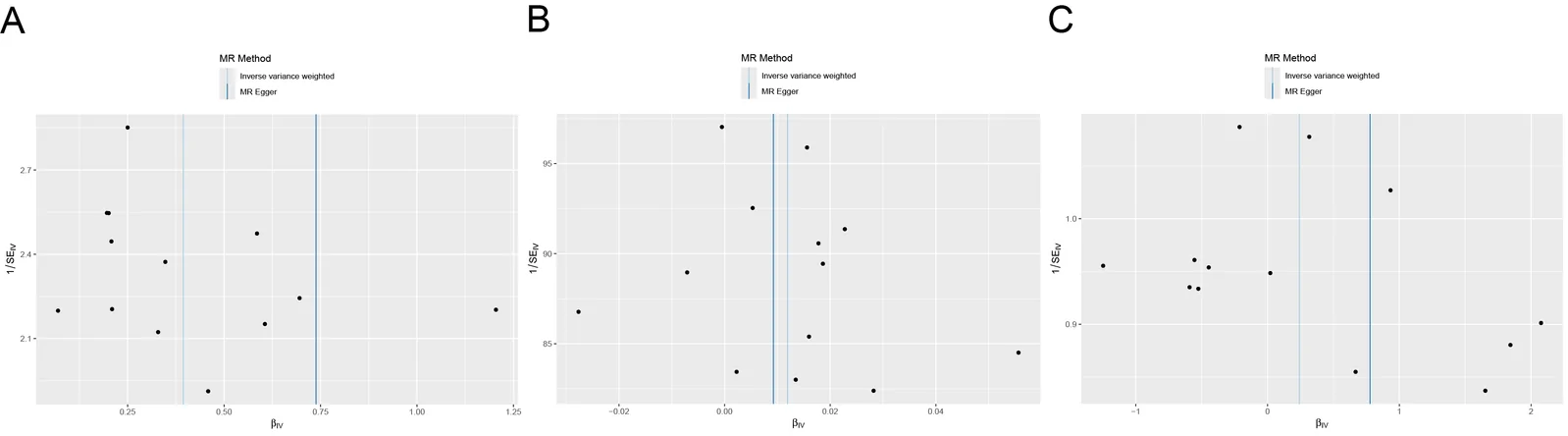
**Funnel plot of MR analysis results**. The (A–C) funnel plot showed the results of inverse variance weighted (IVW) and Mendelian randomisation (MR)-Egger analysis of the causal relationship between antidepressant use and Hashimoto ’s thyroiditis (A), hypothyroidism (B) and thyroid cancer (C).

The funnel plot was generated to evaluate the presence of small-sample effects or potential publication bias by visualising the relationship between the effect estimates of each instrumental variable and its accuracy. The instrumental variable effect estimates of Hashimoto ’s thyroiditis were basically symmetrically distributed on both sides of the IVW method and the MR-Egger estimate, with no marked asymmetry detected. The funnel plot of hypothyroidism showed a similar symmetrical distribution, with instrumental variables of greater precision clustering towards the apex of of the funnel. For thyroid cancer, although the distribution was relatively dispersed due to the dispersion of effect values, the overall symmetry was still good, and no significant evidence of systemic bias was found (Fig. 3). In summary, the funnel plot performance of the three outcomes supports the reliability of the MR analysis results in this study.

### Sensitivity Analysis of Leave-One-Out Method 

**Fig. 4.**
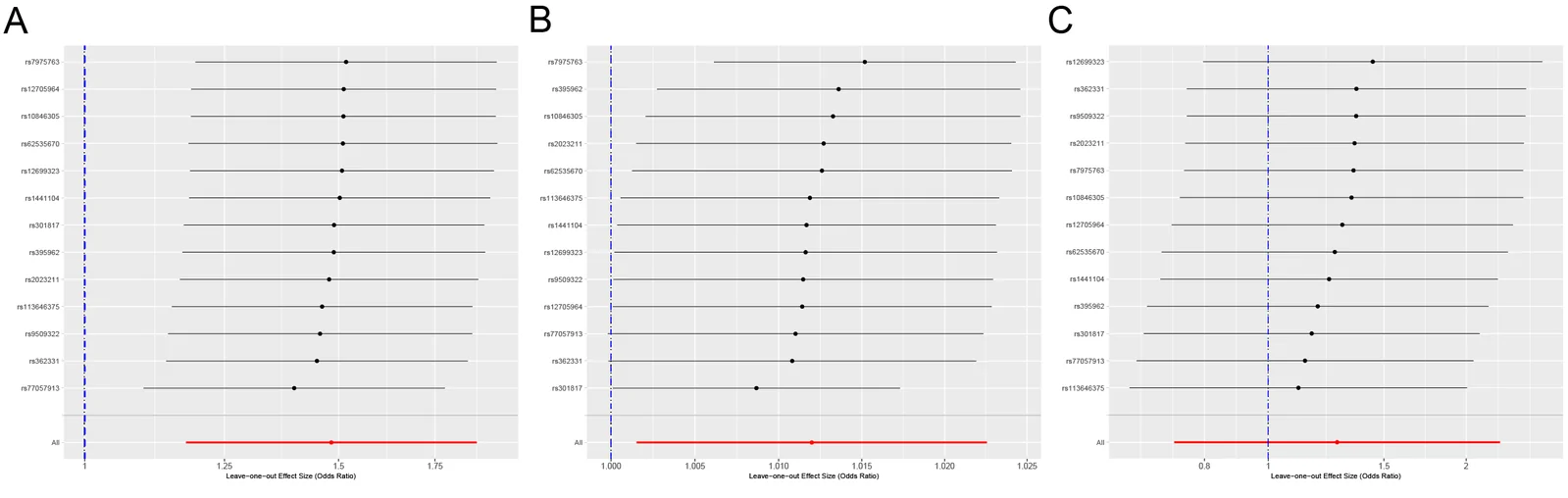
**Leave-one-out sensitivity analyses**. (A–C) Inverse variance weighted (IVW) estimates for the associations of genetically predicted antidepressant use with Hashimoto’s thyroiditis (A), hypothyroidism (B) and thyroid cancer (C) after sequential exclusion of individual single nucleotide polymorphisms (SNPs). The horizontal axis shows the leave-one-out odds ratio (OR) and its 95% confidence interval (CI). The blue dashed line indicates the null value (OR = 1.0).

Leave-one-out sensitivity analysis was performed by sequentially excluding each individual SNPs and recalculating the effect size, to assess whether any influential loci exerted disproportionate impact on the causal estimates (Fig. 4). For Hashimoto’s thyroiditis, the leave-one-out analysis demonstrated that OR estimates remained stable ranging from 1.40 and 1.52 following excluding of any single instrumental variable, with 95% confidence intervals consistently not crossing the null line (OR = 1) and *p*-values retained statistical significance, confirming the robustness of the findings. For hypothyroidism, OR estimates exhibited minimal variation after excluding individual variables, hovering around 1.01 (ranging from 1.009 to 1.015). Although excluding of certain loci resulted in the lower confidence interval boundary to touch the null line due to a marginal loss of statistical power, the magnitude and direction of the overall positive effect did not alter meaningfully, suggesting that this modest positive association is reasonably robust. In the thyroid cancer analysis, effect estimates fluctuated between 1.11 and 1.44 after excluding different SNPs, and all confidence intervals were wide and crossed the null line, further supporting the conclusion that no causal association exists between thyroid cancer and the exposure. Taken together, the leave-one-out results demonstrated that overall effect estimates did not change substantially following removal of any single SNPs, ruling out the possibility that any outlier locus was exerting undue leverage on the causal inference.

### Test of Horizontal Pleiotropy

In this study, the MR-Egger regression intercept test was employed to evaluate whether there is directional pleiotropy that violates the exclusive constraint hypothesis. The results revealed that the intercept *p*-values of Hashimoto’s thyroiditis (*p* = 0.473), hypothyroidism (*p* = 0.902) and thyroid cancer (*p* = 0.661) were all significantly greater than 0.05. This finding indicates that no statistically significant horizontal pleiotropic evidence has been observed, thus supporting the validity premise of Mendelian randomization analysis, that is, the use of antidepressants predicted by genetics is only related to the risk of thyroid disease through the exposure pathway, withs no direct biological pathway operating independent of exposure. This collectively underpins the rigour and robustness of the causal inference conclusions of this study.

## Discussion

This study specifically employed a two-sample Mendelian randomization analysis, utilising genetic variants identified from pooled GWAS data as instrumental variables. From a causal inference perspective, we systematically evaluated the specific associations between antidepressant use and the risk of three thyroid diseases: Hashimoto’s thyroiditis, hypothyroidism, and thyroid cancer. Our findings yielded several key observations: a positive causal association of moderate effect size exists between genetically proxied antidepressant use and the risk of Hashimoto’s thyroiditis, and a positive association—albeit weak but statistically significant—with the risk of hypothyroidism. In contrast, the results indicate that no statistically significant causal effect was observed between antidepressant use and the risk of thyroid cancer.

To substantiate the rigor of the study’s conclusions, we note that the *p*-values for Cochran’s Q-test were all greater than 0.05 in the aforementioned analyses, and the *p*-values for the MR-Egger intercept term were similarly above 0.05. Furthermore, leave-one-out sensitivity analyses confirmed that no single SNPs disproportionately influenced the overall estimates, these findings provide robust evidence that the results of this causal estimation are highly reliable and trustworthy.

The association between antidepressant use and thyroid function has long been a subject of academic attention. In clinical practice, patients receiving antidepressant therapy frequently exhibit fluctuations in TSH levels, abnormalities in thyroid hormone metabolism, and even alterations in thyroid autoantibody titers [27, 28]. However, since depression itself is associated with dysfunction of the HPT axis [7, 8], traditional observational studies have struggled to effectively disentangle the respective contributions of disease-specific effects and pharmacological effects. Furthermore, factors commonly associated with depression, including chronic inflammation, sleep-wake rhythm disturbances, metabolic syndrome and lifestyle changes [29, 30], may act as confounding variables that complicate assessment of the drug-thyroid association. By employing genetic instrumental variables, this study circumvented these phenotypic sources of confounding, providing a new analytical dimension for understanding the causal pathways linking antidepressants to thyroid disease.

Notably, recent Mendelian randomization research hasRecent MR studies have begun to distinguish the contribution of depression itself to thyroid outcomes from that of pharmacological treatment. In a bidirectional two-sample MR study, Zhang *et al*. [31] reported associations of major depressive disorder with hypothyroidism, hyperthyroidism and Hashimoto’s thyroiditis in populations of European ancestry. Their mediation analysis suggested that alcohol intake and antidepressant use partly mediated the pathway from depression to thyroid disease. Fan *et al*. [32] likewise reported associations of recurrent or chronic depression with hypothyroidism (OR = 1.034) and autoimmune thyroiditis (OR = 1.028). These findings suggest that genetic liability to depression may itself contribute to thyroid disease. Accordingly, the associations observed in the present study may reflect both pharmacological pathways and residual genetic liability to the underlying indication, rather than an isolated drug effect.

Among the three outcomes, Hashimoto’s thyroiditis exhibited the most significant causal effect (OR = 1.483), suggesting that antidepressant use may increase the risk of thyroid damage by influencing autoimmune mechanisms. From a pathophysiological perspective, this finding may involve multiple, not entirely independent, biological pathways. The serotonergic system is extensively involved in immune regulation. SSRIs increase 5-hydroxytryptamine (5-HT) concentrations in the synaptic cleft and peripheral circulation by inhibiting the 5-HT transporter, and immune effector cells—including T and B lymphocytes, monocytes/macrophages and dendritic cells—express receptor subtypes such as 5-HT1A, 5-HT2A and 5-HT7 [13, 14]. Sustained increases in peripheral 5-HT might alter T-helper-cell polarisation and cytokine profiles, thereby affecting immune tolerance. Experimental evidence suggests that 5-HT can promote T-helper-cell polarisation towards a T-helper 1 phenotype through receptors including 5-HT2A, increase secretion of pro-inflammatory cytokines such as interferon-γ and enhance natural-killer-cell cytotoxicity [14, 33]. Hashimoto’s thyroiditis is characterised by lymphocytic infiltration, T-helper-1-predominant cytotoxic injury to thyroid tissue, and the production of thyroid peroxidase and thyroglobulin antibodies following abnormal B-cell activation [34, 35]. Crosstalk between the hypothalamic–pituitary–adrenal and HPT axes may provide another pathway. Long-term antidepressant treatment may modify hypothalamic–pituitary–adrenal-axis activity and cortisol rhythms, potentially altering hypothalamic thyrotropin-releasing hormone synthesis and pituitary TSH responsiveness [36]. These mechanisms remain speculative and require direct experimental validation.

The small positive association observed for hypothyroidism (OR = 1.012) is pathophysiologically compatible with the finding for Hashimoto’s thyroiditis, which is a leading cause of hypothyroidism [37]. Progressive autoimmune injury and atrophy of thyroid follicular cells can eventually reduce thyroid hormone synthesis and secretion below the clinical threshold. Therefore, if antidepressants increase the risk of Hashimoto’s thyroiditis by promoting thyroid autoimmune processes, the concomitant increase in the risk of hypothyroidism has a solid pathophysiological basis. The observation that the effect size for hypothyroidism is markedly smaller than that for Hashimoto’s thyroiditis is also biologically plausible: on the one hand, only a portion of patients with Hashimoto’s thyroiditis ultimately progress to clinical hypothyroidism; on the other hand, the etiological spectrum of hypothyroidism is broad, encompassing not only autoimmunity but also iodine deficiency, iatrogenic factors, and congenital defects [37]; therefore, the incremental risk conferred by antidepressants through the single pathway of autoimmunity is effectively “diluted” within the overall effect. Notably, the weighted median method provided independent validation of this association (OR = 1.015, *p* = 0.002); this method can still yield consistent estimates even when up to 50% of instrumental variables are invalid [25], further strengthening. Nevertheless, the IVW effect estimate was very small. The high statistical power afforded by large-sample GWAS data may capture subtle genetic susceptibilities, yet this does not necessarily translate into clinically meaningful harm in practice. Accordingly, considerable caution is warranted when interpreting these findings, and the real-world clinical significance of these results remains to be established through more direct empirical evidence.

No evidence of an association between genetically predicted antidepressant use and thyroid cancer was observed (IVW: *p* = 0.407). Thyroid carcinogenesis, particularly that of papillary thyroid carcinoma, involves molecular events such as activation of the rat sarcoma virus (RAS)/B-Raf proto-oncogene, serine/threonine kinase (*BRAF*)/mitogen-activated protein kinase pathway, rearranged during transfection/papillary thyroid carcinoma (*RET*/PTC) rearrangements and telomerase reverse transcriptase promoter mutations [38]. These drivers differ fundamentally from the mechanisms underlying autoimmune thyroid disease. Although epidemiological studies have reported a higher incidence of papillary thyroid carcinoma among patients with Hashimoto’s thyroiditis [39], whether this relationship is causal or reflects referral and detection biases remains controversial. The Mendelian randomization results of this study do not support the hypothesis that antidepressants increase the risk of thyroid cancer via genetic prediction pathways. This suggests that the effects of antidepressants on the thyroid may be primarily limited to immunomodulation and functional metabolism, and are insufficient to drive the multistep molecular processes involved in tumorigenesis.

This study has several strengths. First, the two-sample MR design is less susceptible than conventional observational studies to reverse causation and some forms of confounding. All F statistics exceeded 10 (mean, 24.12), suggesting a low risk of weak-instrument bias. Second, three outcomes with distinct pathophysiological features—Hashimoto’s thyroiditis (autoimmune), hypothyroidism (functional) and thyroid cancer (neoplastic)—were evaluated within a common analytical framework. This provided a multidimensional overview of the impact of antidepressants on thyroid health, offering greater informational value than analyses focused on a single outcome.Third, the use of IVW, MR-Egger, weighted-median and mode-based methods, together with heterogeneity, pleiotropy and leave-one-out analyses, allowed the consistency and robustness of the estimates to be examined under different assumptions.

Several limitations should be considered. First, the exposure was a binary measure of antidepressant use and could not be stratified by drug class, dose or treatment duration. The estimates therefore represent genetic liability to antidepressant use in the study population and cannot be attributed to a specific agent. Second, all GWAS datasets comprised populations of European ancestry; generalisability to populations with different genetic backgrounds or iodine status remains uncertain. Third, MR estimates reflect lifelong genetic liability to the exposure and may differ in magnitude and temporal pattern from the pharmacological effects of acute or short-term treatment. Fourth, collider bias is possible because variants associated with antidepressant use may capture genetic liability to depression-related traits, such as body mass index (BMI) and smoking, that also influence thyroid disease. Although the MR-Egger intercept and Cochran’s Q tests did not identify significant pleiotropy or heterogeneity, residual bias cannot be excluded, particularly given the small number of instruments (n = 13). Fifth, outcomes defined using diagnostic codes in aggregated GWAS data may be affected by non-differential misclassification, which would generally attenuate MR estimates towards the null. Partial sample overlap between the exposure and outcome GWAS datasets is also possible. Although simulation analyses under a worst-case scenario of complete overlap and strong confounding indicated that the resulting bias remained modest (effect-size bias ≤0.014; Type I error rate ≤~6.0%, **Supplementary Table 2**), residual bias cannot be excluded. Sixth, in the GWAS of Hashimoto’s thyroiditis, the FinnGen component used the general HYPOTHYROIDISM endpoint rather than a Hashimoto’s-specific diagnosis because both phenotypes map to the same FinnGen endpoint (Supplementary Materials provided of Sakaue *et al*. [19]). This creates partial phenotypic overlap with the hypothyroidism outcome. The concern is partly mitigated because the UK Biobank component (220 cases; phecode 245.21) was specific to Hashimoto’s disease, and autoimmune thyroiditis is a major cause of hypothyroidism in iodine-sufficient populations Finland is an iodine-sufficient region, and therefore a substantial proportion of the FinnGen cases are likely attributable to underlying autoimmune thyroiditis, although the exact proportion cannot be determined from the available data. Third, the MR effect estimates for the two outcomes are numerically between Hashimoto’s thyroiditis (OR = 1.483) and hypothyroidism (OR = 1.012), although this does not establish that the GWAS capture distinct genetic signals. Nonetheless, we acknowledge that this phenotype mapping issue represents a genuine limitation, and we have transparently documented it in the Outcome Data section. Future studies would benefit from Hashimoto’s-specific GWAS data with finer phenotypic resolution in the FinnGen cohort.

Future work should prioritise drug-class-stratified MR analyses using emerging pharmacogenomic resources, multivariable MR adjusting for BMI, smoking and genetic liability to depression, and longitudinal cohorts with serial thyroid measurements to evaluate the temporal pattern of any effects. Given the high global prevalence of depression and the continued increase in antidepressant prescribing—particularly among middle-aged and older women, who are at increased risk of Hashimoto’s thyroiditis and hypothyroidism—clarification of these associations has substantial public-health relevance.

## Conclusions

This two-sample MR study provides genetic evidence of potential positive associations between antidepressant use and Hashimoto’s thyroiditis and hypothyroidism, but not thyroid cancer. The findings may support consideration of thyroid-function monitoring during antidepressant treatment, particularly in patients with thyroid autoantibodies or borderline thyroid-function abnormalities. Further studies should evaluate individual drug classes, validate the findings in prospective cohorts and investigate the underlying biological mechanisms to inform individualised prescribing decisions.

## Availability of Data and Materials

All data used in this study are from publicly available GWAS summary statistics. The schizophrenia dataset was obtained from the PGC, lipid traits (LDL-C and TG) from the GLGC and EBI databases, and *LPL* cis-eQTL data from the eQTLGen consortium. No new data were generated in this study. Relevant data can be accessed through the corresponding databases, and analysis code is available from the corresponding author upon reasonable request.

## Supplementary Material

Supplementary material associated with this article can be found, in the online version, at https://doi.org/10.62641/aep.v54i4.2313.
